# Two UV-Sensitive Photoreceptor Proteins, Opn5m and Opn5m2 in Ray-Finned Fish with Distinct Molecular Properties and Broad Distribution in the Retina and Brain

**DOI:** 10.1371/journal.pone.0155339

**Published:** 2016-05-11

**Authors:** Keita Sato, Takahiro Yamashita, Yoshihiro Haruki, Hideyo Ohuchi, Masato Kinoshita, Yoshinori Shichida

**Affiliations:** 1 Department of Biophysics, Graduate School of Science, Kyoto University, Kyoto, Japan; 2 Department of Cytology and Histology, Okayama University Graduate School of Medicine, Dentistry and Pharmaceutical Sciences, Okayama, Japan; 3 Division of Applied Biosciences, Graduate School of Agriculture, Kyoto University, Kyoto, Japan; University of Ferrara, ITALY

## Abstract

Opn5 is a group within the opsin family of proteins that is responsible for visual and non-visual photoreception in animals. It consists of several subgroups, including Opn5m, the only subgroup containing members found in most vertebrates, including mammals. In addition, recent genomic information has revealed that some ray-finned fishes carry paralogous genes of Opn5m while other fishes have no such genes. Here, we report the molecular properties of the opsin now called Opn5m2 and its distributions in both the retina and brain. Like Opn5m, Opn5m2 exhibits UV light-sensitivity when binding to 11-*cis*-retinal and forms a stable active state that couples with Gi subtype of G protein. However, Opn5m2 does not bind all-*trans*-retinal and exhibits exclusive binding to 11-*cis*-retinal, whereas many bistable opsins, including fish Opn5m, can bind directly to all-*trans*-retinal as well as 11-*cis*-retinal. Because medaka fish has lost the Opn5m2 gene from its genome, we compared the tissue distribution patterns of Opn5m in medaka fish, zebrafish, and spotted gar, in addition to the distribution patterns of Opn5m2 in zebrafish and spotted gar. Opn5m expression levels showed a gradient along the dorsal–ventral axis of the retina, and preferential expression was observed in the ventral retina in the three fishes. The levels of Opn5m2 showed a similar gradient with preferential expression observed in the dorsal retina. Opn5m expression was relatively abundant in the inner region of the inner nuclear layer, while Opn5m2 was expressed in the outer edge of the inner nuclear layer. Additionally, we could detect Opn5m expression in several brain regions, including the hypothalamus, of these fish species. Opn5m2 expression could not be detected in zebrafish brain, but was clearly observed in limited brain regions of spotted gar. These results suggest that ray-finned fishes can generally utilize UV light information for non-image-forming photoreception in a wide range of cells in the retina and brain.

## Introduction

Opsins constitute a photoreceptive G protein-coupled receptor family responsible not only for visual photoreception, such as image-forming and color vision, but also for non-visual (non-image-forming) photoreception such as photoentrainment of circadian rhythm, photoperiodism, and pupillary light reflex in animals [1]. Most vertebrates have two types of visual cells in their retinas, rods and cones [2]. It is well known that rod cells express rhodopsin for scotopic vision and cone cells express cone pigments for photopic and color vision. Recent sequencing of vertebrate and invertebrate genomes has revealed that animals express diversified opsin genes in addition to visual pigments [3,4]. These opsins are expressed in retinal interneurons, including horizontal, bipolar, amacrine, and ganglion cells and multiple brain regions, which indicates that these opsins are responsible for non-image-forming photoreceptions.

Opsins identified so far are classified into several groups based on amino acid sequence. Opsin classification correlates well with the diversity of their molecular properties. Among opsin groups, the Opn5 (neuropsin) group has members originally found by using a bioinformatics approach on mouse and human genomes and forms one independent opsin group [5]. Since then, multiple Opn5-related genes have been found from non-mammalian vertebrates, and the corresponding proteins were phylogenetically clustered into some distinct subgroups [6]. Mammals have only one Opn5 gene, Opn5m (mammalian type), and non-mammalian vertebrates have additional Opn5 genes (Opn5L1 and Opn5L2) [7]. The Opn5m gene can be found widely in vertebrates from fish to mammals. Previous reports showed that Opn5m functions as a G_i_-coupled UV-sensitive pigment and that this property is common to Opn5m from various vertebrate species [8–10]. In addition, the analysis of its distribution patterns in birds and mammals revealed that Opn5m is localized to retinal interneurons, including the amacrine and ganglion cells, and several brain regions including the hypothalamus [8–11]. Therefore, it is thought that Opn5m can regulate various non-image-forming photoreceptions in these animals. In fact, avian Opn5m modulates a photoperiodic response in the hypothalamus and mammalian Opn5m photo-entrains the local circadian clock in the retina [11–14]. In this study, we characterized an additional Opn5 paralog found exclusively in ray-finned fishes that is closely related to Opn5m.

## Results

### Genomic characterization of Opn5m2

**Fig 1:**
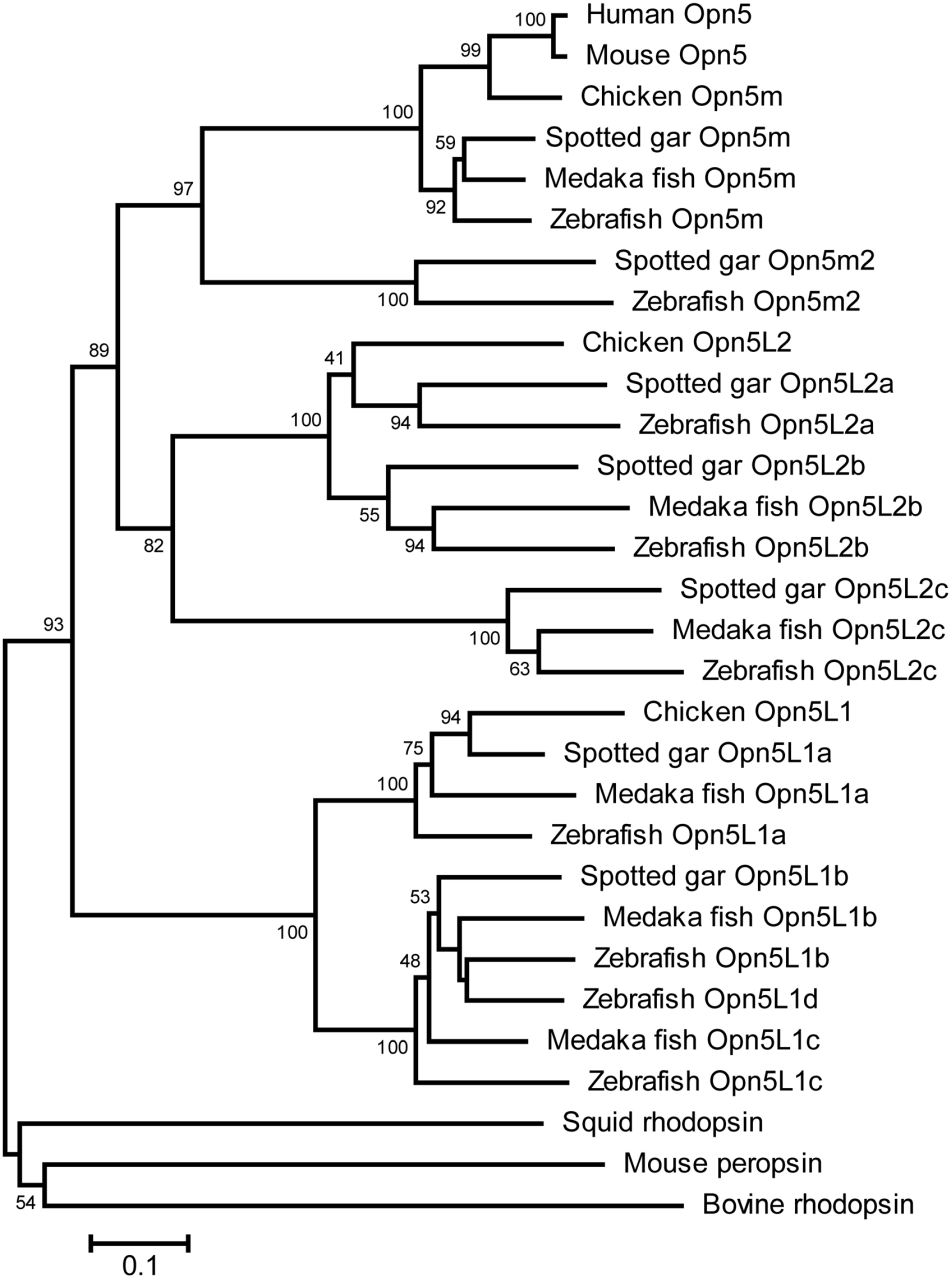
Phylogenetic tree for Opn5 genes. The phylogenetic tree of Opn5 genes was constructed using the Neighbor-Joining method. The percentage of replicate trees in which the associated taxa clustered together in the bootstrap test (1000 replicates) are shown next to the branches. The tree is drawn to scale, with branch lengths in the same units as those of the evolutionary distances used to infer the phylogenetic tree. The evolutionary distances were computed using the Poisson correction method and are in terms of the number of amino acid substitutions per site.

**Fig 2:**
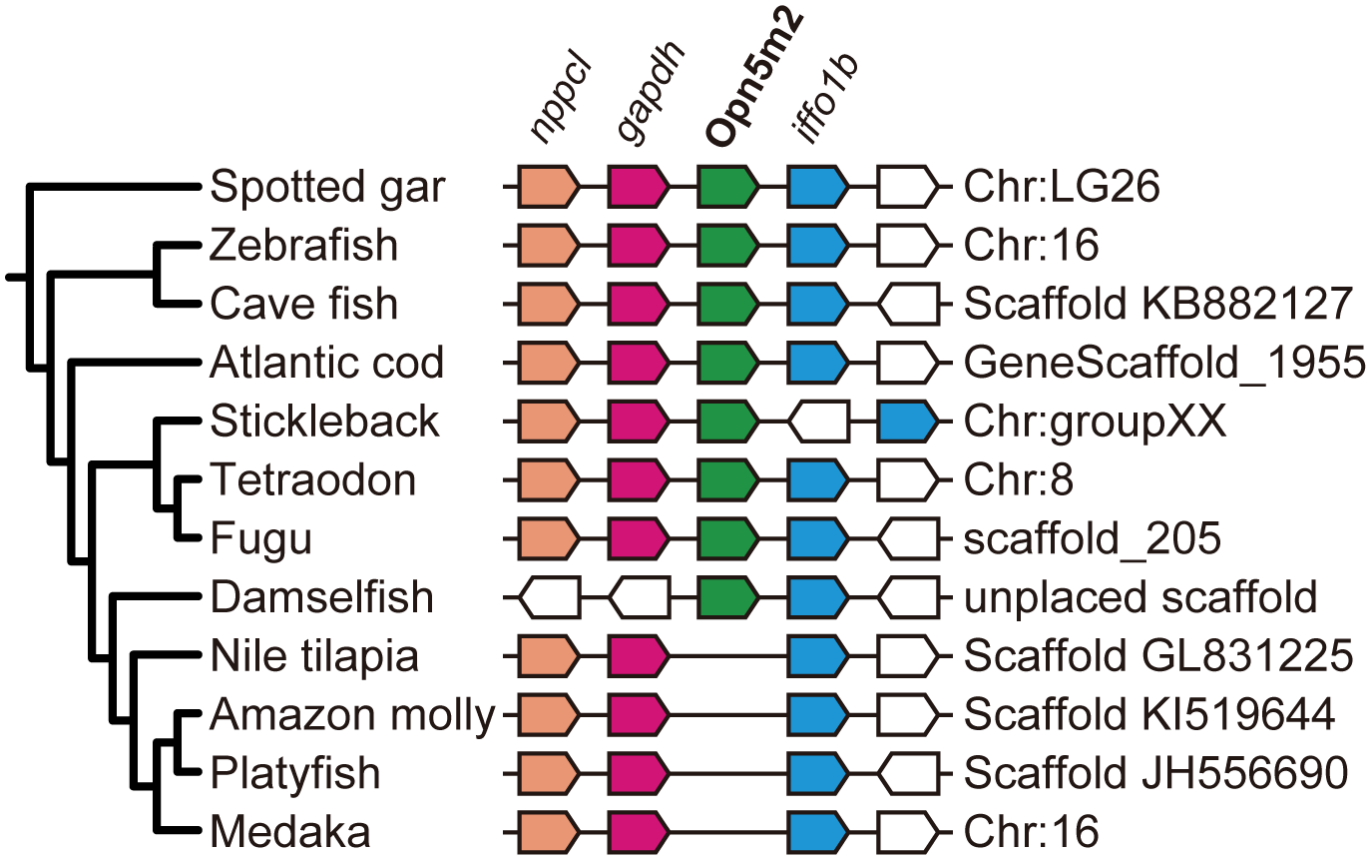
Synteny of Opn5m2 gene in ray-finned fish species. Phylogeny and syntenic orthologues in genomic regions containing the Opn5m2 gene of ray-finned fishes are shown. Phylogenetic relationship of ray-finned fish species was drawn based on Near, et al [17]. Pentagons represent the genes and the direction of the complementary strand. Blue, green, red, and orange pentagons correspond to orthologues of *iffo1b*, Opn5m2, *gapdh*, and *nppcl*, respectively.

A BLAST search using mammalian Opn5m sequences as bait on fish genome databases identified two opsin genes, which are more closely related to Opn5m than other Opn5 subgroups in several fish species. One is phylogenetically clustered with tetrapod Opn5m and thus could be categorized as an Opn5m gene. In contrast, the other was found only in teleostei and holostei and constituted a sister group of Opn5m in the phylogenetic tree of Opn5 group (Fig 1). Therefore, we refer to this gene as Opn5m2. The zebrafish Opn5m2 has average amino acid identities of 46.6 ± 0.7% (n = 6), 35.2 ± 1.3% (n = 10), and 34.9 ± 1.9% (n = 9) to those of Opn5m, Opn5L1, and Opn5L2 in Fig 1, respectively. The amino acid identities between the sequence of zebrafish Opn5m2 and those of the spotted gar Opn5m2, squid rhodopsin, bovine rhodopsin, and mouse peropsin are 68.2%, 34.1%, 23.8%, and 30.9%, respectively. To calculate the amino acid identities, all positions containing gaps in a multiple sequence alignment were excluded (see Materials and Methods). We could not find the Opn5m2 gene in tetrapoda, coelacanthimorpha (coelacanth) or chondrichtyes (elephant shark) genomes. However, we found Opn5m2 genes from deposited nucleotide databases of ray-finned fishes (actinopterygii). Fig 2 shows the synteny of Opn5m2 genes from the genome databases of ray-finned fishes [15–18]. The Opn5m2 gene is located in the conserved synteny, flanked by glyceraldehyde 3-phosphate dehydrogenase (*gapdh*) and intermediate filament family orphan 1b (*iffo1b*) genes. We could not find the Opn5m2 gene from medaka fish, platyfish, amazon molly, or tilapia, although the genomes of these species have a similar arrangement of natriuretic peptide C-like (*nppcl*), *gapdh*, and *iffo1* genes. In the phylogenetic relationship of teleosts, the Amazon molly, platyfish, and medaka fish are classified into one monophyletic group, the cladus Atherinomorpha, while the Nile tilapia is also closely related to them. Hence, these data imply an evolutional scenario in which the common ancestor of ray-finned fishes had already acquired the Opn5m2 gene before the whole genome duplication event in Teleostei, and the common ancestor of Nile tilapia, Amazon molly, platyfish, and medaka fish subsequently lost it (S1A Fig). Alternatively, if the Opn5m2 gene emerged before branching of the Actinopterygii and the Sarcopterygii, the gene would have been independently lost in the ancestor of the Sarcopterygii and specific teleost lineages in Actinopterygii (S1B Fig). Further genomic analyses of vertebrate species will reveal the scenario of when the gene duplication of Opn5m and Opn5m2 occurred in the early evolutionary process of Osteichthyes. To obtain further insight into the physiological relevance of the gene duplication of Opn5m and Opn5m2 in the early evolution of ray-finned fishes, we compared the molecular properties and distribution patterns of these opsins in several fish species.

### Molecular properties of Opn5m2

**Fig 3:**
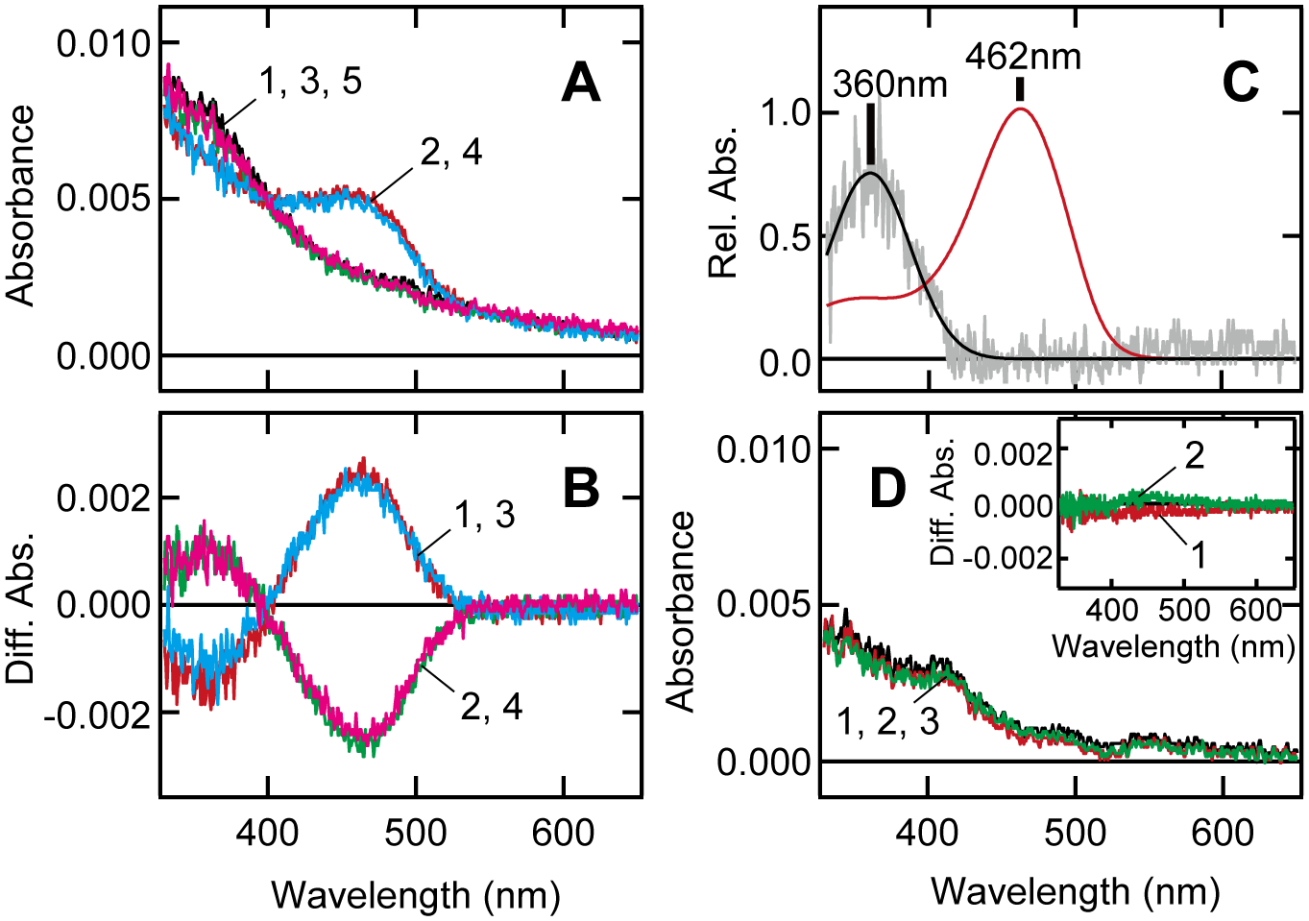
UV-visible absorption spectral properties of zebrafish Opn5m2. A, Absorption spectra of zebrafish Opn5m2 purified after addition of 11-*cis*-retinal. Spectra were recorded in the dark (curve 1, black) and after UV light irradiation (curve 2, red), subsequent yellow light (>480 nm) irradiation (curve 3, green), and following repetition of UV (curve 4, cyan) and yellow light irradiations (curve 5, magenta), respectively. B, Difference spectra calculated based on the spectra in panel A. Curve 1 (red) is the difference spectrum calculated by subtracting curve 2 from curve 1 in panel A. Curves 2, 3, and 4 also show the spectral changes from curve 2 to 3, from curve 3 to 4, and from curve 4 to 5, in panel A, respectively. C, The calculated absorption spectra of zebrafish Opn5m2 in the dark and after UV light irradiation. The absorption spectrum of visible light-absorbing pigment (red curve) was calculated by fitting the visible spectral region of curve 3 in panel B according to the method described in a previous study [8]. The gray curve was acquired by subtracting the best-fitted curve from curve 3 in panel B, which is fitted by a Gaussian function (black curve). The spectra were normalized to the peak absorption of the visible-light absorbing pigment. The calculated absorption maxima were 360 and 462 nm for UV- and visible light-absorbing pigments, respectively. D, Absorption spectra of zebrafish Opn5m2 purified following addition of all-*trans*-retinal. Spectra were recorded in the dark (curve 1, black), after yellow light (>480 nm) irradiation (curve 2, red), and subsequent UV light irradiation (curve 3, green). Inset, Difference spectra calculated by subtracting curve 2 from curve 1 (curve 1, red), and curve 3 from curve 2 (curve 2, green) in panel D.

To examine whether or not Opn5m2 can form a photopigment, we expressed a recombinant protein of zebrafish Opn5m2 in cultured mammalian cell lines in the presence of 11-*cis*-retinal. The UV-visible absorption spectra of recombinant Opn5m2 were recorded after they were solubilized with dodecyl maltoside and purified by affinity column chromatography. The resulting pigment exhibited absorbance in the UV region (Fig 3A). Irradiation with UV light caused the absorption spectrum to shift to the visible region, and subsequent irradiation with a yellow light caused the formation of a spectrum identical in shape with that of the original one. These results indicate that Opn5m2 has two spectral forms, the UV light-absorbing and visible light-absorbing forms, which are interconvertible with each other by light. These spectral characteristics are similar to those of previously reported avian and mammalian Opn5m, strongly suggesting that UV light- and visible light-absorbing forms are 11-*cis*- and all-*trans*-retinal binding forms, respectively. We calculated the absorption spectra of the UV light- and visible light-absorbing forms. The absorption maximum (λ_max_) of the UV light-absorbing form (360 nm) is the same as that of zebrafish Opn5m, whereas λ_max_ of the visible light-absorbing form (462 nm) is ~10 nm blue-shifted compared with that of zebrafish Opn5m (Fig 3C). We subsequently expressed the recombinant protein in the presence of all-*trans*-retinal in cultured cells to determine if Opn5m2 can bind directly to all-*trans*-retinal. Irradiation with yellow or UV light caused no detectable absorption change (Fig 3D). This result showed no formation of a pigment, indicating that Opn5m2 lost the ability to bind directly to all-*trans*-retinal. These observations are in contrast to those previously reported for zebrafish Opn5m, but are apparently similar to those of mammalian Opn5m [10].

### Comparison of Opn5m and Opn5m2 distribution patterns

**Fig 4:**
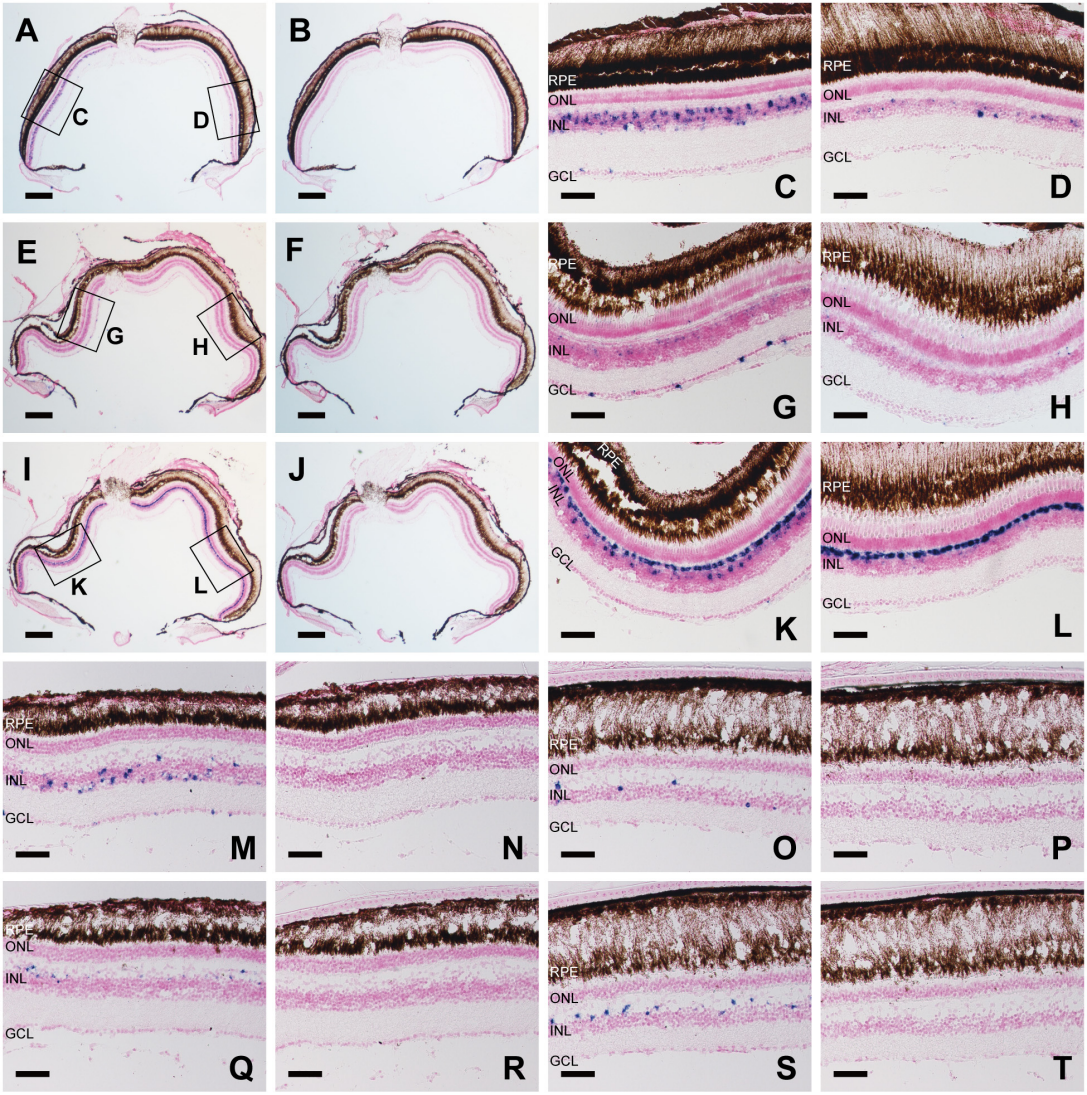
*In situ* hybridization analysis of Opn5m and Opn5m2 mRNA in the retina of medaka fish, zebrafish, and spotted gar. A-D, Detection of medaka fish Opn5m in the retina. Frontal consecutive sections were hybridized with Opn5m antisense (A) and sense (B) probes. Enlarged views of the boxed areas, ventral and dorsal sides, in (A) are shown in (C) and (D), respectively. E-H, Detection of zebrafish Opn5m in the retina. Frontal consecutive sections were hybridized with Opn5m antisense (E) and sense (F) probes. Enlarged views of the boxed areas, ventral and dorsal sides, in (E) are shown in (G) and (H), respectively. I-L, Detection of zebrafish Opn5m2 within the retina. Frontal consecutive sections were hybridized with Opn5m antisense (I) and sense (J) probes. Enlarged views of the boxed areas, ventral and dorsal sides, in (I) are shown in (K) and (L), respectively. M-P, Detection of spotted gar Opn5m within the retina. Ventral regions were hybridized with Opn5m antisense (M) and sense (N) probes. Dorsal regions were hybridized with Opn5m antisense (O) and sense (P) probes. Q-T, Detection of spotted gar Opn5m2. Ventral regions were hybridized with Opn5m antisense (Q) and sense (R) probes. Dorsal regions were hybridized with Opn5m antisense (S) and sense (T) probes. All the sections shown in this figure were counterstained with Nuclear Fast Red. Scale bar: A, B, E, F, I, J, 200 μm; C, D, G, H, K, L, M-T 50 μm

Vertebrate UV light-sensitive opsins characterized so far are categorized into three groups: cone pigment for visual photoreception, parapinopsin and Opn5 for non-visual photoreception. In ray-finned fishes, UV light-sensitive cone pigment is expressed in the retina while parapinopsin is expressed in the pineal gland [19]. We investigated the distribution patterns of UV light-sensitive Opn5m and Opn5m2 in ray-finned fishes. In non-mammalian vertebrates, it is well known that retinal interneurons, ganglion cells and some neurons in the brain have the ability to receive external light signals directly. To determine the precise cellular location of the expression of Opn5m and Opn5m2 in these fish species, we performed *in situ* hybridization on the retina and brain. We previously reported that chicken and mammal Opn5m are distributed in a subset of retinal amacrine and ganglion cells and in several brain regions, including the pineal gland and hypothalamus. We went on to investigate the expression patterns of Opn5m and Opn5m2 in the retinas and brains of three different fish species: zebrafish, medaka fish, and spotted gar. Zebrafish and medaka fish belong to the Teleostei, but medaka fish lacks the Opn5m2 gene. Spotted gar belongs to the Holostei and has both Opn5m and Opn5m2 genes. Because the Holostei group branched from the Teleostei lineage in the phylogeny of ray-finned fishes before the whole-genome duplication event, it is interesting to analyze the distribution of Opn5-related expressions for understanding the evolution of the UV light-sensing system in ray-finned fishes. Through the analysis of the hybridization signal of Opn5m and Opn5m2 in the retinas of these fishes, we found that the signal intensities were significantly altered in the dorsal-ventral orientation in the retina. First, we analyzed the pattern of Opn5m mRNA expression in the medaka fish retina. Hybridization signals for Opn5m were relatively denser in the ventral region rather than in the dorsal region (Fig 4A and 4B). In the ventral retina, the transcript of Opn5m was expressed densely in the population throughout the inner nuclear layer (INL), except for horizontal cells, and in a small number of ganglion cells (Fig 4C, S2A and S2B Fig). In the dorsal retina, hybridization signals were observed in fewer subsets of cells in the INL (Fig 4D, S2C Fig). Next, we compared the expression patterns of zebrafish Opn5m and Opn5m2 in the retina. Opn5m hybridization signals were detected only in the ventral side of the retina (Fig 4E and 4F). We observed strong signals in a sparse population of ganglion cells and weak ones in the INL (Fig 4G, 4H and S2D Fig). Strong Opn5m2 hybridization signals were detected in the horizontal cells of both the dorsal and ventral regions (Fig 4I and 4J), while signals in the dorsal retina were denser than those in the ventral retina (S2E and S1F Figs). Additionally, we also observed signals in bipolar or Müller cells of the ventral retina (Fig 4K, 4L and S2E Fig). Moreover, we successfully detected Opn5m and Opn5m2 mRNA in the spotted gar retina. Hybridization signals were sparse for spotted gar Opn5m in the dorsal retina (Fig 4O and 4P) and abundant in the ventral retina (Fig 4M, 4N and S2G Fig). Specific expression signals were detected in a variety of cells in the INL, which is similar to those of medaka fish Opn5m. In contrast, Opn5m2 expression was relatively strong in the dorsal retina, especially in the outer edge of the INL (Fig 4Q–4T, S2H Fig), which is similar to the zebrafish Opn5m2 expression pattern. However, spotted gar Opn5m2 was expressed in a smaller population of horizontal cells, or cell types of the INL than those in zebrafish retina. In summary, the expression of Opn5m was more prominent in the ventral retina than in the dorsal retina of these fishes. Additionally, Opn5m was distributed predominantly on the inner side of the INL and in the ganglion cell layer, which is generally consistent with the expression pattern of Opn5m in avian and mammalian retinas. Furthermore, Opn5m2-positive cells were more abundant in the dorsal retina of zebrafish and spotted gar, which is in contrast to the expression pattern of Opn5m. Expression was detected predominantly in the outer side of the INL, which is also in contrast to that of Opn5m.

**Fig 5:**
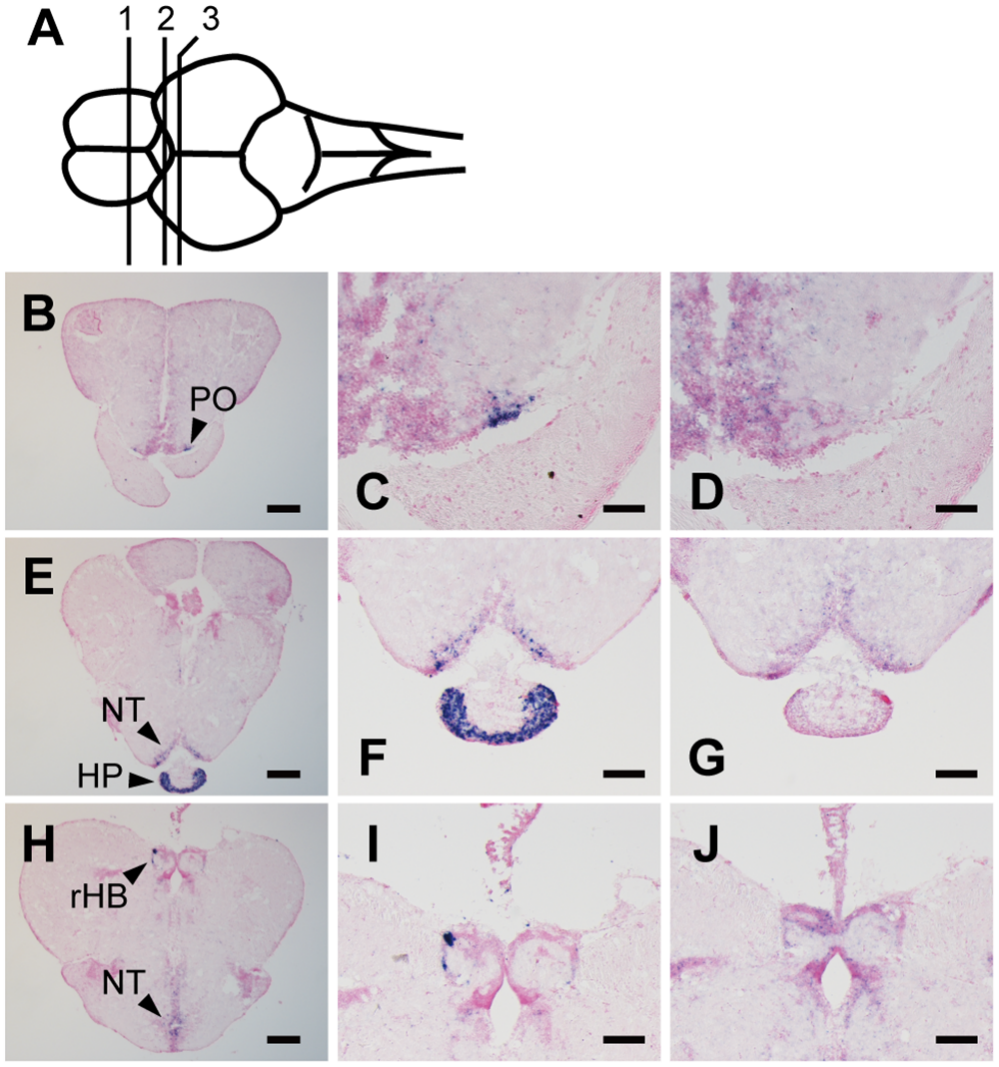
Distribution of Opn5m mRNA within medaka fish brain. A, Schematic drawing of medaka fish brain, dorsal view. Numbered lines indicate the positions of cross sections shown in B-J. B-J, Localization of medaka fish Opn5m in the brain. Expression signals were detected within frontal sections cut along lines 1 (B), 2 (E) and 3 (H). Sections were hybridized with Opn5m antisense (B, C, E, F, H, I) and sense (D, G, J) probes. Enlarged views of regions around preoptic area in panel B, pituitary in panel E, and habenula in panel H are shown in panels C, F, and I, respectively. Panels D, G, and J show the consecutive tissue sections to C, F, and I hybridized with Opn5m sense probe, respectively. All sections shown in this figure were counterstained with Nuclear Fast Red. Scale bar: B, E, H, 200 μm; F, G, I, J, 100 μm; C, D, 50 μm.

**Fig 6:**
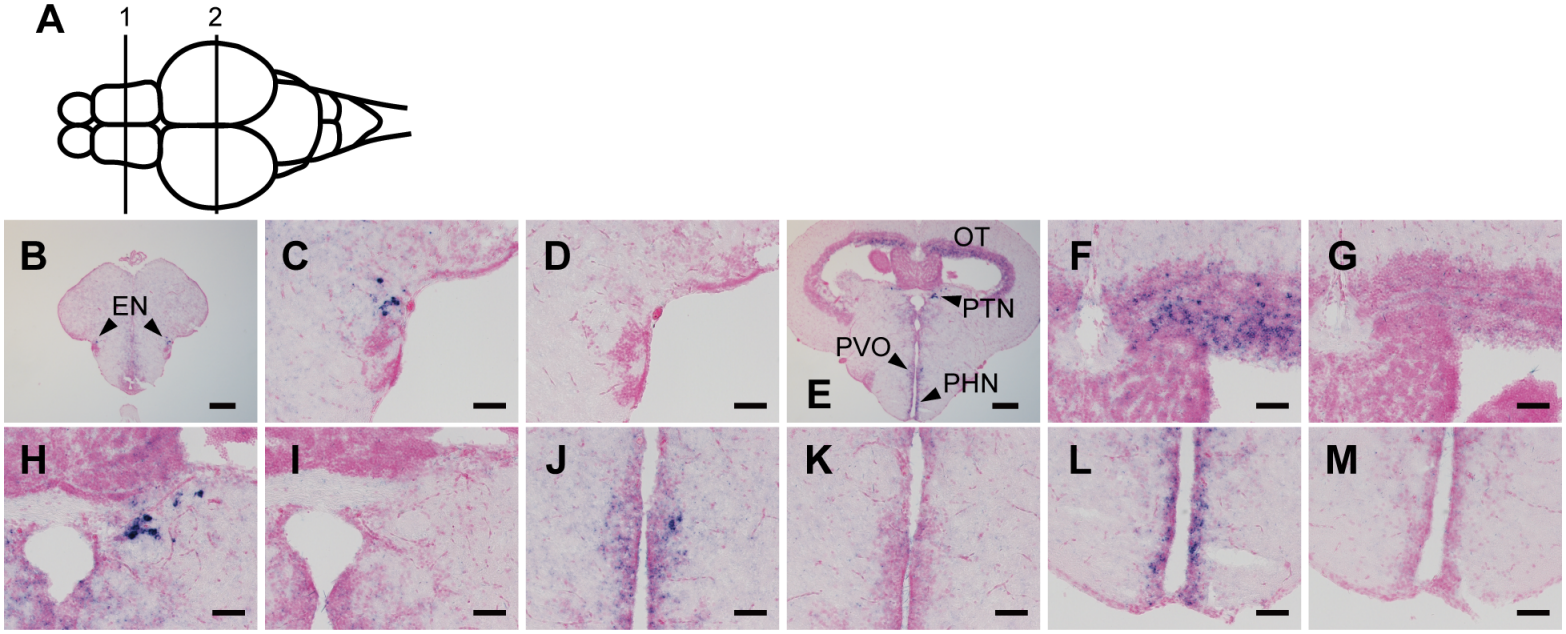
Distribution of Opn5m mRNA within zebrafish brain. A, Schematic drawing of zebrafish brain, dorsal view. Numbered lines indicate the positions of cross sections shown in B-M. B-M, Localization of zebrafish Opn5m within the brain. Expression signals were detected within frontal sections cut along lines 1 (B), and 2 (E). Sections were hybridized with Opn5m antisense (B, C, E, F, H, J, L) and sense (D, G, I, K, M) probes. Enlarged views of regions around entopeduncular nucleus in panel B, optic tectum, pretectal nucleus, paraventricular organ, and periventricular nucleus in panel E are shown in panels C, F, H, J, and K, respectively. Panels D, G, I, K, and M show consecutive tissue sections to C, F, and I hybridized with Opn5m sense probe, respectively. We could not detect any hybridization signals for Opn5m2 in the zebrafish brain. All sections shown in this figure were counterstained with Nuclear Fast Red. Scale bar: B, E, 200 μm; C, D, F-M, 50 μm.

**Fig 7:**
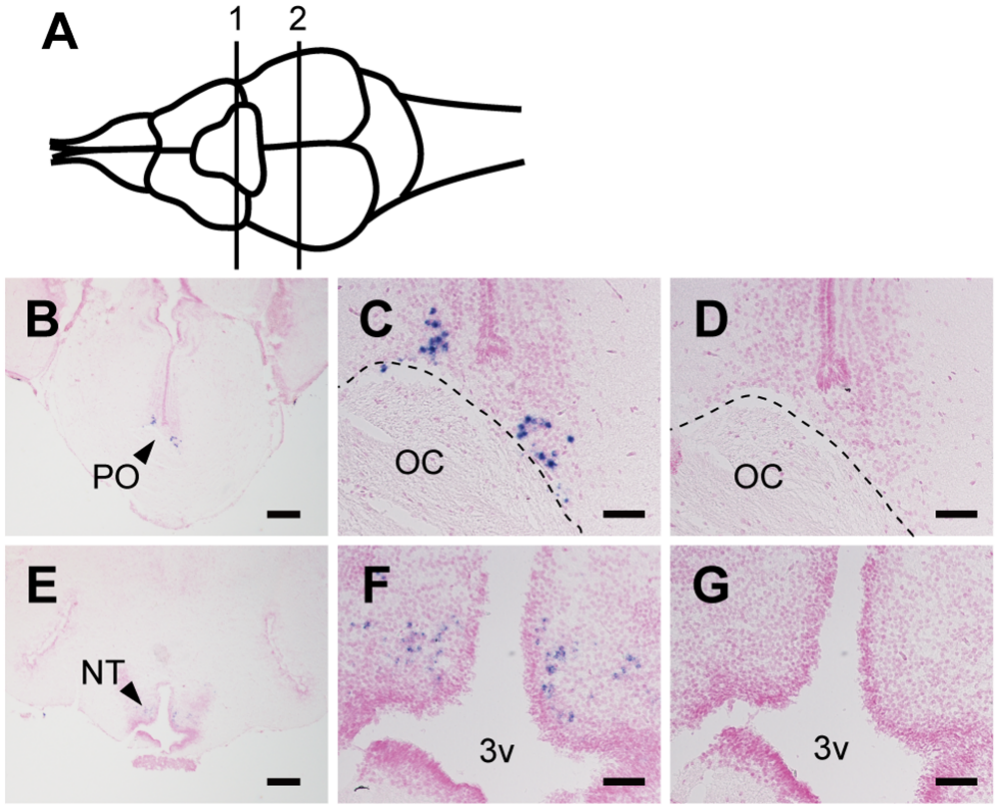
Distribution of Opn5m and Opn5m2 mRNA within spotted gar brain. A, Schematic drawing of spotted gar brain, dorsal view. Numbered lines indicate the positions of cross sections shown in B-G. B-D, Localization of spotted gar Opn5m within the brain. Expression signals were detected within the frontal section cut along line 1. Sections were hybridized with Opn5m antisense (B, C) and sense (D) probes. Enlarged view of the region around the optic chiasm in B is shown in panel C. Panel D shows the consecutive tissue section to C hybridized with Opn5m sense probe. The broken line indicates the border between optic chiasm and brain parenchyma. E-G, Localization of spotted gar Opn5m2 in the brain. Expression signals were detected in the frontal section cut along line 2. Sections were hybridized with Opn5m2 antisense (E, F) and sense (G) probes. Enlarged view of the region around third ventricle in panel E is shown in F. Panel G shows the consecutive tissue section to F hybridized with Opn5m sense probe. All sections shown in this figure were counterstained with Nuclear Fast Red. Scale bar: B, E, 200 μm; C, D, F, G, 50 μm.

Next, we examined the expression patterns of Opn5m and Opn5m2 in the brain of the three fishes. In the medaka fish brain, we detected the expression of Opn5m in several brain regions, including the preoptic area, tuberal nucleus, pituitary, and right habenula (Fig 5, S3A–S3D Fig). In the zebrafish brain, hybridization signals of Opn5m were detected in the endopeduncular nucleus, optic tectum, pretectal nucleus, paraventricular organ, and periventricular nucleus (Fig 6, S3E–S3I Fig). However, we could not detect significant mRNA expression for zebrafish Opn5m2 in the brain. In the spotted gar brain, the hybridization signals of Opn5m and Opn5m2 were detected in quite small numbers of cells in the brain. There is limited information about the brain atlas of spotted gar. Thus, based on the correspondence with the zebrafish brain atlas, Opn5m is expressed dorsally in the preoptic area along the optic chiasm, and Opn5m2 is weakly expressed in the tuberal nucleus along the third ventricle (Fig 7, S3J and S3K Fig). No hybridization signals could be detected in the other brain regions (Figs 5, 6 or 7). These results show that the expression patterns of orthologous Opn5 genes are highly diversified within ray-finned fishes.

## Discussion

In the present study, we investigated the molecular properties and the expression patterns of an additional Opn5 paralog, Opn5m2, found exclusively in ray-finned fishes and closely related to Opn5m. Opn5m2 can form a UV light-sensitive pigment by the incorporation of 11-*cis*-retinal, which is shared with the spectral sensitivity of fish Opn5m. However, Opn5m2 does not form a photo-pigment by direct binding to all-*trans*-retinal, which is in contrast with the binding preference of retinal isomers in fish Opn5m. Direct binding of all-*trans*-retinal to opsin can form a G protein activation state without light, increasing noise component of light-dependent signaling. Mammalian Opn5m has lost the ability to bind to all-*trans*-retinal due to a single alanine-to-threonine mutation at position 168 (in the bovine rhodopsin numbering system) during the course of molecular evolution [10]. However, Opn5m2 contains an alanine residue at position 168 just like in non-mammalian Opn5m. This indicates that amino acid residue(s) at other position(s) control the binding specificity of Opn5m2 to retinal isomers, and that independent evolutionary events have enabled mammalian Opn5m and fish Opn5m2 to work as a UV light sensor without noise of direct all-*trans*-retinal binding.

Our analysis of the distribution patterns of Opn5m and Opn5m2 showed that these opsins are localized in retinal interneurons and ganglion cells with a dorsal-ventral gradient of expression levels. It was reported that two cone pigments, UV- and green-sensitive ones, were expressed in a dorsal-ventral gradient in mouse retina [20,21]. Similar expression patterns were also reported in four green-sensitive cone pigments in zebrafish retina [22–24]. However, there are no reports on the dorsal-ventral alteration of the expression level of non-image-forming opsins in the retina. Therefore, we are the first to demonstrate that non-image-forming opsins are expressed in a dorsal-ventral gradient in the retina. Ventral and dorsal retinas would mainly receive downwelling and upwelling light, respectively. Many fish species have UV light–sensitive cone pigments, suggesting that UV light is an important transmitted signal in aquatic environments. Generally, downwelling light originates from the sun, whereas upwelling light is due to light that is reflected or scattered underwater. Thus, we can speculate that Opn5m2 in dorsal retina, which has a high signal-to-noise ratio for light signaling, is appropriate for detecting weak reflected or scattered light. Retinal interneurons and ganglion cells of the Teleostei express several opsins, such as melanopsin, VA opsin, and TMT opsin, and exhibit an intrinsic light response [25–31]. These non-image-forming opsins are all sensitive to visible light, whereas Opn5m and Opn5m2 are uniquely sensitive to UV light [29,32–36]. UV light reception by Opn5m and Opn5m2 would therefore modify the visual photoreception system in a cooperative manner as secondary inputs, and also contribute directly to non-visual functions in retinal interneurons and ganglion cells with visible light inputs by other non-image-forming opsins.

The analysis of expression patterns in the brain showed that Opn5m-positive cells are localized not only in the hypothalamus but also in other brain regions of ray-finned fishes, some of which are responsible for reproductive activities in vertebrates. In the medaka fish and spotted gar brains, Opn5m was distributed in the preoptic area, which is consistent with the expression pattern of Opn5m in the mammalian brain [10]. The expression patterns of several genes in the preoptic area of both mammals and fishes indicate sexual dimorphism [37,38]. This suggests that Opn5m plays a conserved role in vertebrate brains, probably in the regulation of sexual behavior or reproductive activity. Opn5m was also expressed abundantly in the medaka fish pituitary gland. Thus, medaka fish Opn5m may directly regulate the production and secretion of pituitary hormones such as gonadotropin [39]. Moreover, Opn5m was localized to the tuberal nucleus of medaka fish and in the periventricular nucleus of zebrafish. Previous reports showed that kisspeptin is expressed in the tuberal nucleus and periventricular nucleus of medaka fish and zebrafish [40–42]. Kisspeptin plays a crucial role in regulating the activity of the GnRH neuron in mammals [43], whereas knockouts of kisspeptin1 or 2 do not impair reproductive activity in zebrafish [44]. Recent reports showed that kisspeptin2 cells in the periventricular nucleus of zebrafish have fibers that form a wide network projecting to several brain regions [45]. Thus, Opn5m may be related to the unknown physiological function of kisspeptin in medaka fish and zebrafish. As seen in retinal interneurons, it has been reported that melanopsin, VA opsin and TMT opsin are also expressed in multiple teleost brain regions, such as the optic tectum and hindbrain [29,46,47]. By using a variety of non-image-forming opsins, the teleost brain would be capable of sensing a wide range of wavelengths from UV to visible light for the modulation of long-term activities, such as neuroendocrine activities, and short-term activities, such as sensory inputs. In contrast to Opn5m, the expression level of Opn5m2 in the brain was quite low. We could not identify Opn5m2-positive cells in the zebrafish brain and could detect them only in a cluster of tuberal nuclei of the spotted gar. The molecular properties of Opn5m2 showed that the function of Opn5m2 requires the molecular system to supply the 11-*cis*-retinal, which may lead to quite a small number of Opn5m2-positive cells in the brain due to a shortage of 11-*cis*-retinal.

In conclusion, we showed that Opn5m and Opn5m2 diverged by gene duplication early in the evolution of ray-finned fishes, exhibit common UV light-sensitivity, and exhibit different affinities to retinal isomers. In addition, the distribution patterns of Opn5m and Opn5m2 in three ray-finned fishes revealed that these opsins are localized in multiple retinal interneurons and ganglion cells with a dorsal-ventral gradient of expression levels, and also in several brain regions, including the hypothalamus. Therefore, in contrast to only a small number of UV light-sensing visual cells for visual photoreception, a wide range of cells in the retina and brain have the potential to respond to UV light. This finding suggests the importance of UV light information for various non-visual photoreceptions in ray-finned fishes.

## Materials and Methods

### Animals and ethics statement

Adult zebrafish (*Danio rerio*: ~3 cm) and juvenile spotted gars (*Lepisosteus oculatus*: ~7 cm) were purchased from Shimizu Laboratory Supplies Co., Ltd., Kyoto, Japan, and Nippon Aquarium Co., Ltd., Tokyo, Japan, respectively. They were euthanized and dissected immediately after they were brought into our laboratory. The medaka fish (*Oryzias latipes*) strain d-rR was maintained and bred at Kyoto University. They were kept under a light/dark cycle of 14/10 h at 25°C. Adult medaka fishes (~3 cm) were chosen for the experiment. Fishes were euthanized by immobilization using MS222 and immediately decapitated thereafter. The use of animals in these experiments was in accordance with guidelines established by the Ministry of Education, Culture, Sports, Science and Technology of Japan. The protocols in this paper were approved by the Animal Care and Use Committee of Kyoto University (permit number: 26–71).

### Search for Opn5-related genes and isolation of their cDNAs

The clones of ray-finned fish Opn5m and Opn5m2 were subject to BLAST searches against Ensemble or NCBI databases by using mammalian Opn5m sequences. Although tetraodon Opn5m2 was not annotated in these databases, exons of orthologous genes were found between the gapdh and iffo1b genes in tetraodon genome through blast search using zebrafish Opn5m2 as bait. Accordingly, cDNA sequence of tetraodon Opn5m2 was manually organized from predicted exons. To express recombinant proteins and conduct *in situ* hybridization, cDNAs of Opn5m and Opn5m2 (NCBI accession number; XM_004083639, AY493740, XM_005157939, XM_006626015, XM_015337843) were cloned from total RNA of eyes and brain of medaka fish, zebrafish, and spotted gar.

### Phylogenetic analysis and synteny mapping

Multiple amino acid sequences were aligned using MAFFT [48]. The phylogenetic tree was inferred by using MEGA6 software [49]. The analysis involved 30 amino acid sequences. All positions containing gaps and missing data were excluded. There were a total of 252 positions in the final dataset. A synteny map was constructed using Genomicus [15,16] except for damselfish whose map was manually organized based on the data in NCBI database (NW_007578555).

### Preparation of recombinant proteins

Detailed procedures to prepare recombinant Opn5 proteins were previously reported [10]. In short, the cDNA encoding zebrafish Opn5m2 tagged with the epitope sequence of the anti-bovine rhodopsin monoclonal antibody Rho1D4 (ETSQVAPA) at the C terminus was introduced into the mammalian expression vector, pCAGGS. The plasmid DNA was transfected into the HEK293S cell line using the calcium phosphate method. After a day of incubation, 11-*cis*-retinal or all-*trans*-retinal was added to the medium (final retinal concentration, 5μM). After additional incubation for 1 day in the dark, opsin-expressing cells were collected. The pigments were extracted with a buffer containing dodecyl maltoside and purified using Rho1D4-conjugated agarose.

### Spectrophotometry

Absorption spectra were recorded using a Shimadzu UV2450 spectrophotometer and an optical cell (width, 2 mm; light path, 1 cm) according to the previous study [10]. An optical cell-holder was connected to a Neslab RTE-7 temperature controller, which kept the sample temperature at 0 ± 0.1°C. The sample was irradiated with light from a 1 kW tungsten halogen lamp (Rigaku Seiki) that had been passed through a glass cutoff filter (Y50 and UV-D36).

### Tissue sample preparation

After eyes and brains were dissected from juvenile spotted gars (~7 cm), adult zebrafish (~3 cm) and adult medaka fish (~3 cm), they were fixed overnight in PBS-buffered 4% (zebrafish and medaka fish) or 8% (spotted gar) PFA. Tissues were subsequently immersed in 20% sucrose in PBS overnight for cryoprotection and were frozen in a deep freezer in OCT compound (tissue tech). Frozen tissues were sliced into 16 μm sections and were attached to glass slides (MAS-GP typeA coated glass slide, Matsunami Glass Co.,Ltd.). Slides were stored in a dry chamber at -20°C.

### *In situ* hybridization

*In situ* hybridization on tissue sections was performed according to the previously described protocol [10] with a slight modification. Digoxigenin-labeled RNA probes were synthesized from Opn5 open reading frame cDNAs inserted into either pBluescript KS(+) or pTA2 vector (TOYOBO Co., LTD.) using either T7 or T3 RNA polymerase. Tissue sections on slide glasses were successively immersed in PBS-buffered 4% PFA for 15 min, methanol for 30 min, PBS for 5 min, Tris buffer (50mM Tris-HCl, 10 mM NaCl, pH 7.2) containing 0.2 ug/ml proteinase K for 15 min, PBS for 5 min, PBS-buffered 4% PFA for 15 min, DEPC-treated water for 2 min, acetylation buffer (0.27%(v/v) acetic anhydride, 0.1 M triethanolamine, pH 8.0) for >10 min, and PBS for 5 min. Then, slides were transferred into hybridization buffer (0.75 M NaCl, 75 mM sodium citrate, 0.2 mg/mL yeast tRNA, 0.1 mg/mL heparin sodium, 1x Denhardt’s solution, 0.1% (v/v) Tween, 0.1% (w/v) CHAPS, 5mM EDTA, 50% (v/v) formamide) and incubated for 3 hours at 65°C. After that, sections on slide glasses were incubated with digoxigenin-labeled RNA sense or antisense probe diluted with hybridization buffer in a moist chamber box for about 40 hours at 65°C. After hybridization, they were successively immersed in SSC buffer (0.15M NaCl, 15 mM sodium citrate, pH 7.0) containing 50% formamide for 15 min and for 1 hours at 65°C, one-fifth diluted SSC buffer for 1 hour at 65°C, and MABT (100 mM maleate, NaCl, 0.1% Tween 20, pH 7.5) for three times 30 min at R.T. After washing, the sections were incubated with blocking buffer (1% BSA, 10% sheep normal serum and 1% Triton-X100 in PBS) for 30 min. After that, they were incubated with anti-digoxigenin Fab fragment conjugated with alkaline phosphatase in a moist chamber box overnight at 4°C. The slides were subsequently washed three times with MABT for 30 min, and twice with alkaline phosphatase reaction buffer (100 mM Tris-HCl, 50 mM MgCl_2_, 100 mM NaCl, 0.1% Tween 20, pH 9.5). Finally, they were immersed in alkaline phosphatase reaction buffer containing NBT and BCIP for color development.

## Supporting Information

S1 Fig Possible evolutionary scenarios for the acquisition and loss of Opn5m2 gene. (TIF)

S2 Fig High-power field microscopic images of *in situ* hybridization in fish retinas (600x magnification). (A) Medaka fish Opn5m in the INL of the ventral retina. (B) Medaka fish Opn5m in the GCL of the ventral retina. (C) Medaka fish Opn5m in the dorsal retina. (D) Zebrafish Opn5m in the ventral retina. (E) Zebrafish Opn5m2 in the ventral retina. (F) Zebrafish Opn5m2 in the dorsal retina. (G) Spotted gar Opn5m in the ventral retina. (H) Spotted gar Opn5m2 in the dorsal retina. Scale bar: 10 μm.
(TIF)

S3 Fig High-power field microscopic images of *in situ* hybridization in fish brains (600x magnification). (A) Medaka fish Opn5m in preoptic area. (B) Medaka fish Opn5m in tuberal nucleus. (C) Medaka fish Opn5m in pituitary gland. (D) Medaka fish Opn5m in right habenula. (E) Zebrafish Opn5m in entopeduncular nucleus. (F) Zebrafish Opn5m in optic tectum. (G) Zebrafish Opn5m in pretectal nucleus. (H) Zebrafish Opn5m in paraventricular organ. (I) Zebrafish Opn5m in periventricular nucleus. (J) Spotted gar Opn5m in preoptic area. (K) Spotted gar Opn5m2 in tuberal nucleus. Scale bar: 10 μm.
(TIF)

## Abbreviations

RPE: retinal pigment epithelium
ONL: outer nuclear layer
INL: inner nuclear layer
GCL: ganglion cell layer
PO: preoptic area
NT: tuberal nucleus
HP: hypophysis (pituitary gland)
rHB: right habenula
EN: entopeduncular nucleus
OT: optic tectum
PTN: pretectum nucleus
PVO: paraventricular organ
PHN: periventricular nucleus
OC: optic chiasm
3v: third ventricle
tris: tris(hydroxymethyl)aminomethane
