# Supplementary figures and images for: Two UV-Sensitive Photoreceptor Proteins, Opn5m and Opn5m2 in Ray-Finned Fish with Distinct Molecular Properties and Broad Distribution in the Retina and Brain
Source: PLoS One. 2016 May 11;11(5):e0155339. doi: 10.1371/journal.pone.0155339 (PMC4864311; doi:10.1371/journal.pone.0155339)

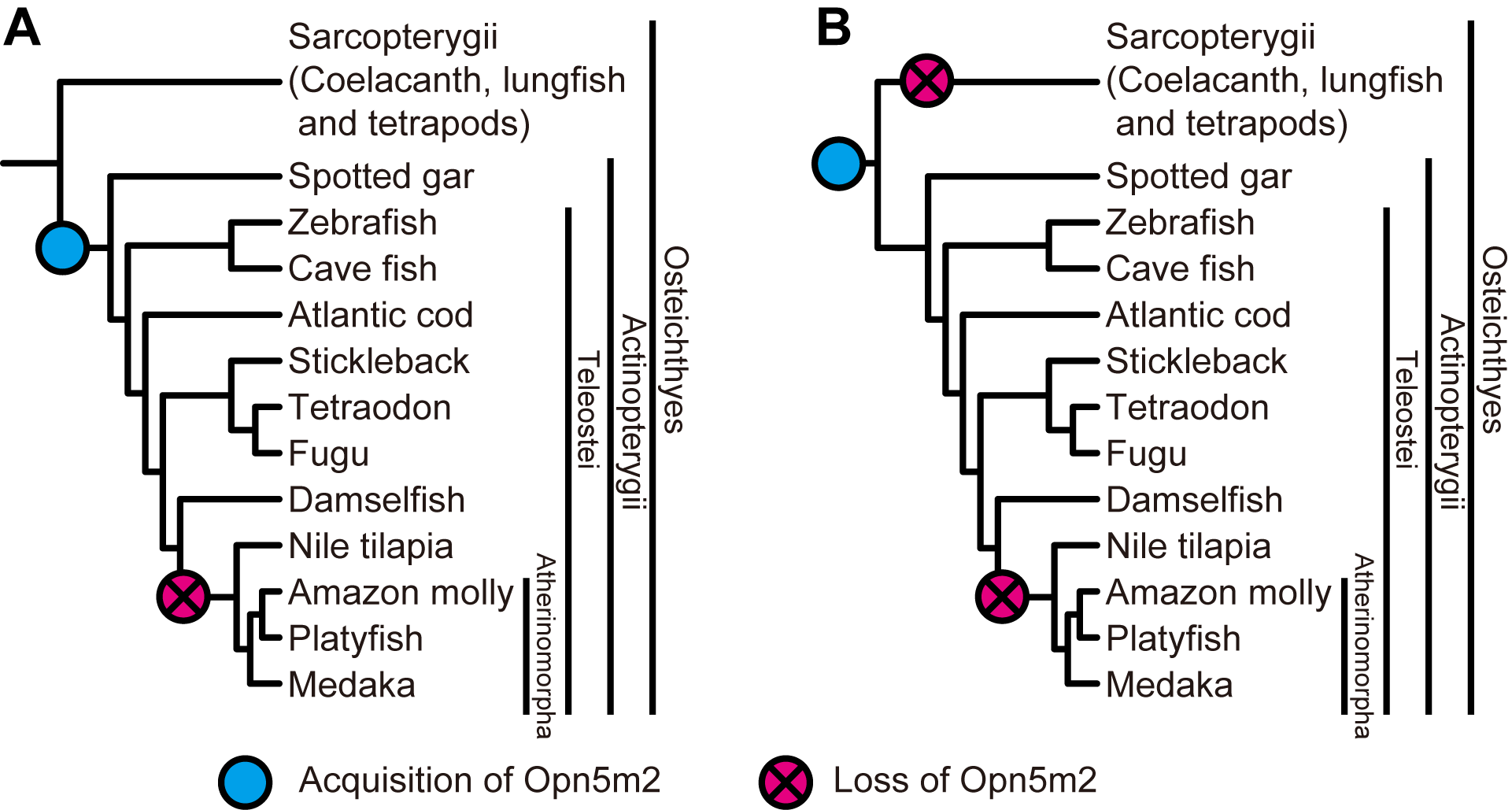

Supplement: S1 Fig — (TIF) [file pone.0155339.s001.tif]

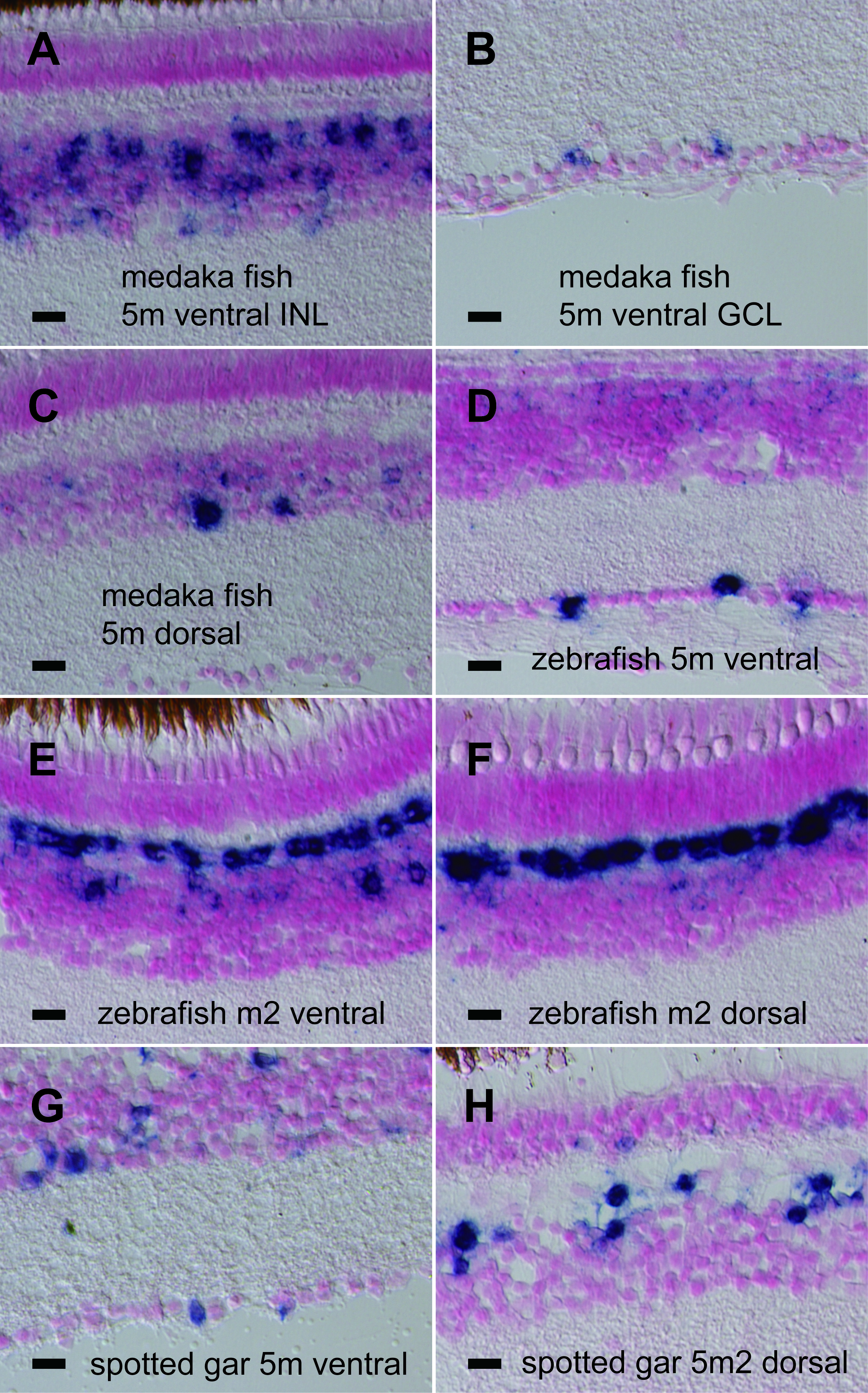

Supplement: S2 Fig — (A) Medaka fish Opn5m in the INL of the ventral retina. (B) Medaka fish Opn5m in the GCL of the ventral retina. (C) Medaka fish Opn5m in the dorsal retina. (D) Zebrafish Opn5m in the ventral retina. (E) Zebrafish Opn5m2 in the ventral retina. (F) Zebrafish Opn5m2 in the dorsal retina. (G) Spotted gar Opn5m in the ventral retina. (H) Spotted gar Opn5m2 in the dorsal retina. Scale bar: 10 μm. (TIF) [file pone.0155339.s002.tif]

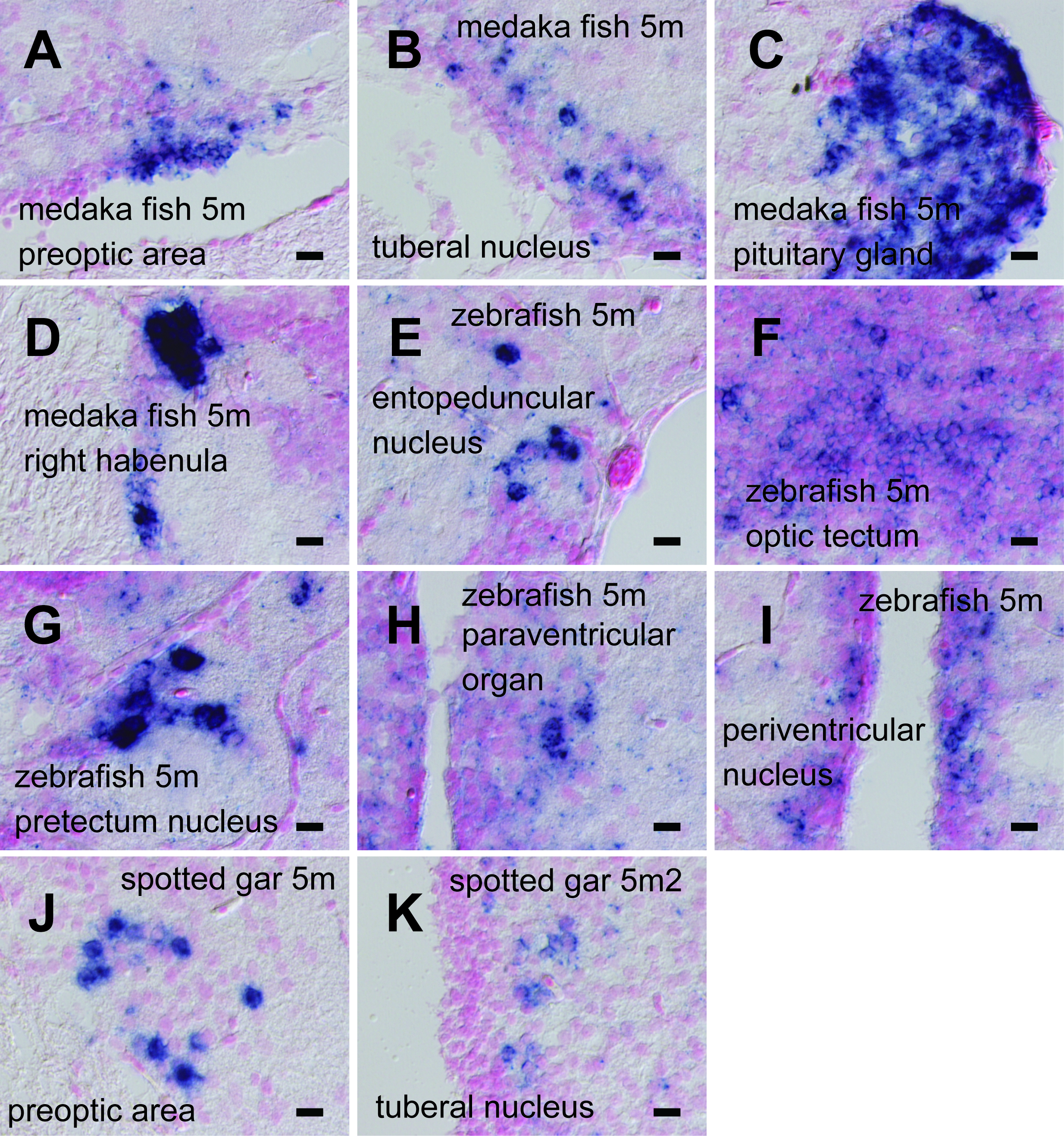

Supplement: S3 Fig — (A) Medaka fish Opn5m in preoptic area. (B) Medaka fish Opn5m in tuberal nucleus. (C) Medaka fish Opn5m in pituitary gland. (D) Medaka fish Opn5m in right habenula. (E) Zebrafish Opn5m in entopeduncular nucleus. (F) Zebrafish Opn5m in optic tectum. (G) Zebrafish Opn5m in pretectal nucleus. (H) Zebrafish Opn5m in paraventricular organ. (I) Zebrafish Opn5m in periventricular nucleus. (J) Spotted gar Opn5m in preoptic area. (K) Spotted gar Opn5m2 in tuberal nucleus. Scale bar: 10 μm. (TIF) [file pone.0155339.s003.tif]
